# Machine Perfusion and the Pancreas: Will It Increase the Donor Pool?

**DOI:** 10.1007/s11892-019-1165-y

**Published:** 2019-07-10

**Authors:** Karim Hamaoui, Vassilios Papalois

**Affiliations:** Department of Surgery, Imperial College London, London, UK

**Keywords:** Pancreas, Machine perfusion, Organ preservation, Donor pools, Extended criteria donor, Viability assessment, Graft preconditioning

## Abstract

**Purpose of Review:**

Pancreas transplantation enables complete patient independence from exogenous insulin administration and increases both patient survival and quality of life. Despite this, there has been a decline in pancreas transplantation for the past 20 years, influenced by changing donor demographics with more high-risk extended criteria (ECD) and donation after cardiac death (DCD) donors. This review discusses whether the advent of machine perfusion (MP), if extended to the pancreas, can increase the pool of suitable donor organs.

**Recent Findings:**

Hypothermic and normothermic MP, as forms of preservation deemed superior to cold storage for high-risk kidney and liver donor organs, have opened the avenue for translation of this work into the pancreas. Recent experimental models of porcine and human ex-vivo pancreatic MP are promising. Applications of MP to the pancreas however need refinement—focusing on perfusion protocols and viability assessment tools.

**Summary:**

Emerging research shows pancreatic MP can potentially offer superior preservation capacity, the ability to both resuscitate and manipulate organs, and assess functional and metabolic organ viability. The future of MP will lie in organ assessment and resuscitation after retrieval, where ultimately organs initially considered high risk and unsuitable for transplantation will be optimised and transformed, making them then available for clinical use, thus increasing the pool of suitably viable pancreata for transplantation.

## Introduction: Current State of Pancreas Transplantation

Diabetes mellitus is one of the fastest growing chronic diseases in the world with an estimated 422 million people, or 8.5% of the world’s population, suffering from diabetes in 2014 [1]. In the UK, there are 4.7 million persons with diabetes and a further 1.1 million people who have diabetes and remain undiagnosed. Overall, 10% of people with diabetes have type 1 diabetes [2] and the prevalence of type 1 diabetes has doubled in the past 20 years increasing the burden on health services and society [3]. Insulin revolutionized the treatment of type 1 diabetes when it was first discovered in 1921 and was shown to be lifesaving. Minimizing hyperglycaemia has been recognized as an important goal in management with tight control of blood glucose levels beneficial in preventing progression of subsequent secondary diabetic complications [4, 5]. Despite the significant advances in insulin delivery, exogenous insulin treatment still cannot provide the suitable equivalent physiological control to prevent the chronic development of diabetic complications [3, 6]. The only demonstrated cure to date that reliably enables complete patient independence from exogenous insulin administration, and increasing both patient survival and quality of life, is whole-organ pancreas transplantation.

Pancreas transplantation however is not an immediate lifesaving procedure; as such in the majority of cases, it is jointly undertaken in patients with diabetes who also require renal transplantation [7], either in a combined procedure or separately soon after the kidney transplant, as these patients already require immunosuppression. Any beneficial value gained from pancreas transplantation alone, such as sustained euglycaemia without the need for exogenous insulin, must be weighed against the risks of the operative procedure itself and the additional requirement for long-term and potentially nephrotoxic immune suppression in patients with stable renal function [8]. Pancreatic transplantation thus may be considered to be a group of three separate clinical entities [9]: simultaneous pancreas and kidney transplant (SPK), pancreas after kidney transplant (PAK), and pancreas transplant alone (PTA), each of which is characterised by its own indications, risks and outcomes in terms of patient survival, graft survival, and effects on quality of life and secondary complications of diabetes.

Since the first pancreas transplant in 1966 at the University of Minnesota [10] by Kelly and Lillihei, 50,000 patients with diabetes have been transplanted in more than 200 centres worldwide [6].

24,484 pancreas transplants have been performed in the USA, and 4656 in the Eurotransplant region in the past 20 years [11•]. In the UK, 2537 pancreas transplants were performed during this period with 1966 between 2008 and 2018 across 8 UK transplant centres [12].

Most recent registry data report that, in the UK, there were 189 patients on the national pancreas transplant waiting list, with 185 transplants undertaken in 2017/2018 [12]. In the same period in the USA, 1492 patients were added to the pancreas transplant waiting list and a total of 1002 pancreas transplants were performed, while 457 patients were awaiting a pancreas transplant, with 200 performed in the Eurotransplant region in 2018 [13, 14].

## Current Outcomes in Pancreatic Transplantation

Over the past 50 years, significant progress and development in surgical technique, immunosuppression strategies, and both donor and recipient management have yielded both evolving and impressive outcomes. The most demonstrable success of pancreas transplantation remains most evident in the SPK group. SPK is the most common type of pancreas transplant and is performed in a patient with diabetes and typically eGFR < 20 ml/min or having dialysis that is undergoing kidney transplantation. In PAK, a diabetic kidney transplant recipient receives a pancreas to minimize the morbidity associated with diabetes and prevent the development of new diabetic nephropathy in the transplanted kidney. PTA is performed selectively in patients with diabetes complicated by frequent, severe metabolic complications such as hypoglycaemic unawareness, diabetic ketoacidosis, or hyperglycaemia that requires hospital admission. Worldwide, national organizations have different patient criteria, indications, and contra-indications for pancreas transplant listing (including BMI limits and restrictions to degree of pancreatic failure [C-peptide levels]) [15–17].

In the USA, of 1002 transplants performed in 2017, 83% were SPKs, with the remaining 213 transplants split almost equally between PTA and PAK; similar rates were evident in Europe (83% SPK, and 17% PTA/PAK) [13, 14]. In the UK, there was a higher rate of SPK transplants at 89% of patients in 2017/2018; the remaining 11% underwent PTA [12]. PAK is not routinely undertaken in the UK.

In the USA, where more than 1000 pancreas transplants are performed every year, 5-year patient survival is 93% for SPK, 91% for PAK, and 78% for PTA recipients, respectively. Early pancreatic graft failure is 9.2%, 7.3%, and 7.1% for PAK, PTA, and SPK, respectively over the past 3 years, while 5-year graft survival is 73% for SPK, 65% for PAK, and 53% for PTA. The 10-year kidney graft survival in SPK is 66% and is comparable with that of deceased donor kidney transplants in the USA at 47%, while kidney graft survival after PAK is 97.1%, 83.9%%, and 52.9% % at 1, 5, and 10 years, respectively [13].

The most recent 1- and 5-year data from the UK for patient survival for SPK recipients is 98% and 88%, and graft survival 89% and 79% respectively; similarly, for PTA patient, survival is 97% and 78%, and graft survival 88% and 52% respectively [12]. Similar patient and graft survival rates are reported by Eurotransplant [14].

## Proven Benefits of Pancreas Transplantation

The Diabetes Control and Complications Trial (DCCT) [5] showed tight blood glucose control depicted by target glycated haemoglobin (HbA1C) levels of less than 7% in participants with type 1 diabetes lowered the incidence of diabetic retinopathic, neuropathic, and nephropathic lesions by 47%, 60%, and 39% respectively. It is reasonable to suggest that pancreas transplantation in patients with diabetes would benefit patients in relation to progression of secondary diabetic lesions and prolong survival.

Reports have suggested that pancreas transplantation with a kidney can prevent, delay the progression of, and reverse diabetic nephropathy in the kidney grafts of SPK recipients [18]. Furthermore, progression of neuropathy and retinopathy is often delayed after pancreas transplantation, with evidence of improved patient-reported quality of life [19].

Despite these findings, such improvement in glycaemic control and benefit on diabetic lesions with pancreas transplantation must be balanced with the need for immunosuppression and the associated potential nephrotoxic risks of these regimes. As of yet, no randomized controlled trial (RCT) has compared SPK vs kidney transplantation alone, or PTA vs intensive insulin therapy. Multiple single-centre reports and registry analyses however suggest a survival benefit compared with isolated kidney transplantation in recipients with diabetes and kidney impairment [18–20]. The potential impact and risk however of pancreas transplantation on patient survival and kidney function in patients with diabetes but preserved renal function (i.e. PTA) have yet to be determined.

## Current Activity and Decline

Despite ongoing activity and the proven demonstrable success of the procedure and benefits to patients, there has been a significant decline in pancreas transplantation over the past 20 years [21, 22].

In a recent analysis of all data from UNOS, the UK Transplant Registry, and Eurotransplant, there was evidence of this decline [11•]. In both the USA and the Eurotransplant region from 1997, there was a successive expansion in the numbers of transplants undertaken—4.1% and 1.7% annually in the regions respectively. This growth peaked in 2004 with 1484 performed in the USA (5.1 per million population [pmp]) and 282 (2.4 pmp) in Europe, followed by a decline of 2.9% and 1.8% annually in activity between 2004 and 2016 respectively. In slight contrast in the UK, there was rapid and sustained growth in transplant activity from 1997 which lasted until 2009, with annual growth of 60.2% equating to a peak of 225 transplants per year. Since then, activity has remained relatively static with growth declining by a marginal 1.0% per annum until the last reported figures for 2017.

## Explanations for the Decline in Activity Are Multiple

Multiple explanations for the decline have been suggested [11•, 21–23]. There is continued debate regarding the precise role of whole-organ vascularized pancreas transplantation given the evolution of medical treatment options available for diabetes, including islet cell transplantation, and thus may temper referral from nephrologists, endocrinologists, and diabetologists. Despite emerging evidence of good short and long patient and graft outcomes that demonstrates the significant improvements in patient selection, surgical technique, and immunosuppression strategies, these results may not be widely accepted outside the transplant community.

There have also been significant changes in donor demographics over the past two decades. In the UK for example, the numbers of deceased donors increased between 2008 and 2018 while the proportion of those donors classified as DCD increased from 32 to 39% in this period. More than a third of deceased donors are now aged more than 60, an increase from 20 to 36% between 2008 and 2018, with the proportion of donors aged more than 70 years increasing from 5 to 14% in this period. More donors are now clinically obese (BMI > 30) with the proportion increasing from 20 to 28% in the last 10 years. Finally, the proportion of deceased donors arising from death after trauma has fallen from 13 to 3% in this same time period [12]. The expansion of the DCD cohort has occurred largely as an effort to address the growing organ shortage and the demand on waiting lists.

Increasingly, due to the demographic shifts, more donors can be classified as extended criteria donors (ECD: older, high BMI (> 30 kg/m^2^) or hemodynamic instability peri-procurement [24]). A compound effect of these shifts is a low pancreatic utilization rate, with only 60% of pancreata offered for transplantation from DBD donors actually retrieved, and only 30% of those DBD pancreata that are offered being transplanted. Utilization in DCD donors is lower still at 48% of those offered and only 23% of all offered DCD pancreata transplanted [25]. In the UK, the poor rate of conversion from potential donors to a transplant has been associated with concerns regarding donor history, high rates of pancreatic retrieval injury, anxiety over the visual appearance of the retrieved graft related to fat content [26], and the perception that DCD and ECD donor pancreata are at higher risk of delayed graft function (DGF) and surgical complications. This is despite growing evidence of equity in patient and graft survival with judicious use of DCD vs DBD donor pancreata [26, 27].

Similar demographic shifts have been noted in the USA [28], and discard rates for pancreata recovered remain high, with 72.7% of grafts from donors age ≥ 50 years not utilized. Discard rates are directly correlated with increasing donor BMI, with over 80% of pancreata from donors BMI ≥ 35 kg/m^2^ discarded in 2017. Furthermore, there is a reluctance to utilize DCD donor grafts, comprising less than 3% of the organs transplanted [13]. Integration of the pancreatic donor risk index (PRDI)—an algorithmic metric to predict postoperative outcomes—into donor selection criteria, of which DCD is a component, is a likely factor in the low utilization rate of DCD donors in this setting [29].

Despite data that demonstrate acceptable patient and graft survival post pancreas transplantation, the potential risk for morbidity related to perioperative complications and their sequelae (graft thrombosis, pancreatitis) on subsequent graft and patient outcomes has perhaps hindered clinician aggressiveness to pursue both SPK and PAK transplantation for their patients [28].

## Current Strategies to Expand the Pool

Various strategies have been suggested to facilitate the expansion of the donor pool and the supply of suitably viable organs [23, 30]. With more use of DCD donor organs with lengthier warm ischaemia time (WIT), and ECD donors of increasing age, the effect of co-morbidities and ischaemic damage on these organs is increased with the potential for inferior function and survival after transplantation. This potential concern has probably tempered the expansion of pancreas transplantation the most. Maintaining allograft viability using simple static cold storage (SCS) in the face of the increasing numbers of ECD and DCD organs that are being used worldwide in efforts to expand donor pools has most likely reached the limits of basic SCS preservation.

In this context, dynamic developments in machine perfusion (MP) preservation in kidney and liver transplantation, in the form of both hypothermic machine perfusion (HMP) and normothermic machine perfusion (NMP), offer potential for application to pancreas preservation.

HMP is based on the concept of preserving the organ in a ‘better environment’ [31]. Similar to SCS, HMP slows down metabolism, reducing oxygen requirements and ATP depletion by circulating a cold preservative solution through the organ via pulsatile or non-pulsatile flow, theoretically providing nutrients, and facilitating washout out of toxic metabolites and free radicals. NMP is the controlled reperfusion of organs with perfusate at normothermic temperatures with the provision of oxygen replicating near physiological parameters. A recent pan-European expert group highlights that interventions that address the problem of ischaemia-reperfusion injury are likely to find key roles in clinical practice in the years to come [23].

### HMP

**Fig. 1:**
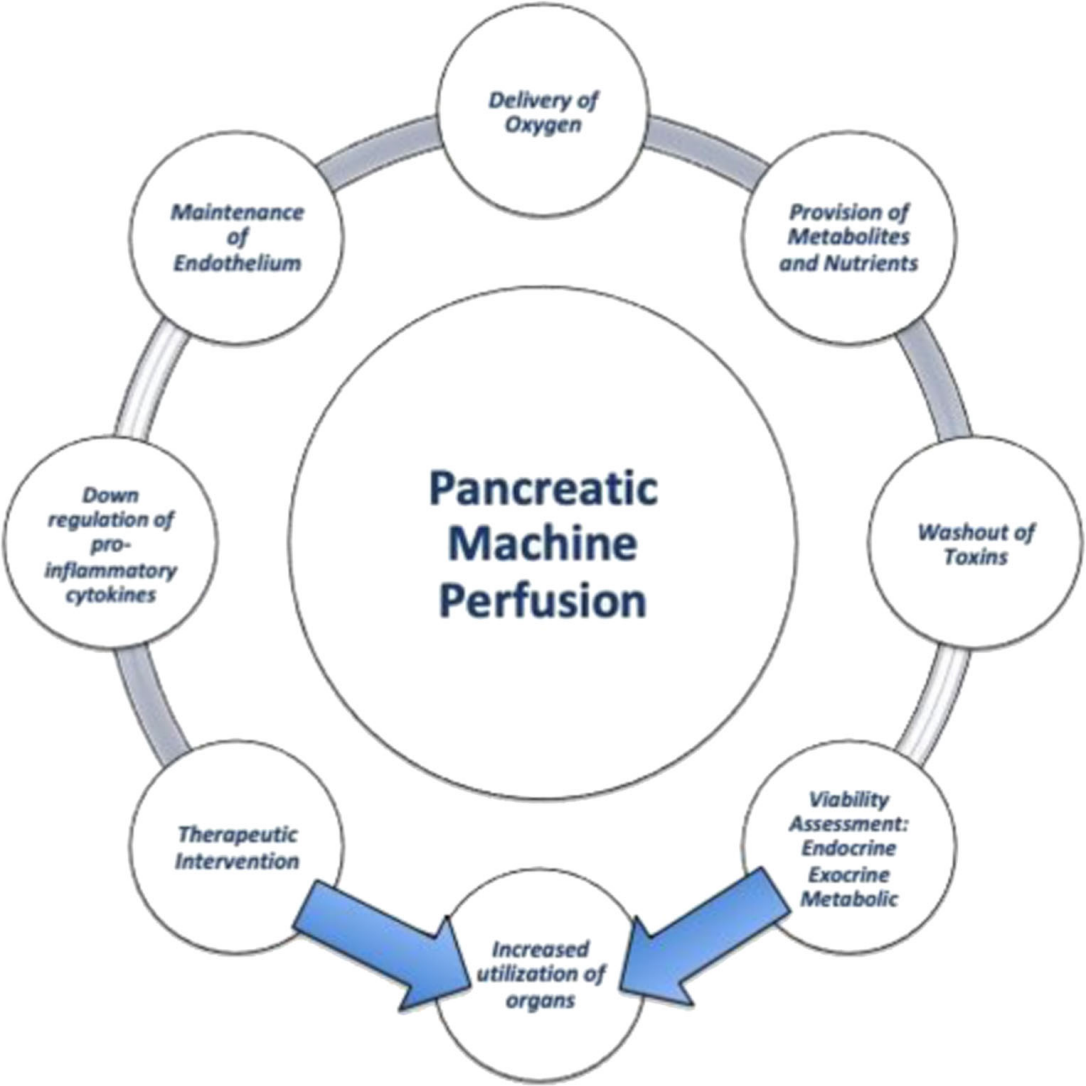
Proven and theoretical benefits of machine perfusion compared with static cold storage in pancreas preservation

HMP use has been demonstrated to have several advantages over SCS in improving allograft viability based on the concepts of hypothermic reconditioning [32] (see Fig. 1). It facilitates the delivery of oxygen and nutrients as well as removal and ‘washout’ of toxic metabolites, and it has been shown to decrease cellular apoptosis, markers of reperfusion injury, expression of pro-inflammatory cytokines, and levels of interstitial fibrosis and tubular atrophy [33]. Evidence suggests that HMP acts by preserving allograft energy metabolites, acting to suppress programmed cell death and promote autophagy, and limiting endothelial activation that upon reperfusion would potentiate a greater inflammatory response [34]. It also enables a degree of organ viability assessment using perfusion parameters and perfusate analysis [33]. The Machine Preservation Trial from Eurotransplant was the first large RCT comparing kidney HMP with SCS and demonstrated benefit of HMP in reducing delayed graft function [35], primarily in ECD and DBD vs DCD donors [36], with suggestions of longer-term graft survival [37], though this has been questioned [38]. The largest benefit of HMP is seen in organs with less than 10 h of cold ischaemia time (CIT), with CIT remaining a significant risk factor for DGF; identifying the potential for HMP in the optimising kidneys with short CIT [39]. In liver preservation, the first few clinical series over the past decade of liver HMP have demonstrated the feasibility and safety of this technique [40, 41]. This strategy is currently being investigated compared with SCS in several RCTs (ClinicalTrials.gov Identifier: NCT03031067, NCT01317342).

### NMP

NMP is designed to resuscitate the organ after a period of SCS and is carried out for a short period while the recipient is being prepared for transplantation. Once NMP is complete, NMP graft is temporarily placed back in SCS until transplantation. Experimental evidence suggests it enables through the provision of oxygen and nutrients the restoration of cellular energy reserves and ATP.

The clinical application of NMP has recently emerged. In a small clinical trial in the UK by Hosgood et al. [42•] using a series of ten declined DCD kidneys, 60 min of normothermic oxygenated perfusion allowed ‘resuscitation’ and assessment of these kidneys using a quality assessment score (composite of macroscopic appearance, mean renal blood flow, total urine output). Of these ten kidneys, five were determined to be of sufficient viability (score < 3) to warrant transplantation. There were no instances of acute rejection, and one episode of delayed function with successful follow-up to 6 months. A large RCT (ISRCTN15821205) is currently underway and exploring the benefit of NMP in DCD category III/IV kidney transplantation in the UK [43].

The goals in the development of dynamic preservation are to allow optimisation and assessment of organs prior to transplantation. Areas of remaining uncertainty include the precise cohort of donor organs these modalities are most beneficial for, which modality is optimal (cold vs normothermic), or what criteria can be used to judge viability.

## Pancreatic Machine Perfusion

The preservation method of choice in whole pancreas transplantation is currently SCS. Despite the definite benefits of transplantation, the shortage of suitable organs is a predominant factor in limiting whole-pancreas transplantation. This, combined with the increased use of ECD donors, has led several groups to attempt to translate the success of MP with kidneys to the maintenance and assessment of the quality of pancreatic grafts [33].

It can be assumed that, because renal HMP preceded other forms of dynamic organ preservation, attempts to apply HMP to other organs have routinely been based on modifying renal protocols. There are however a number of reasons why such protocols cannot be directly applied to the pancreas, first identified by the Minnesota Group [44]. The main physiological difference with the pancreas is its low flow and pressure environment, and thus, MP has the capacity to damage the fragile vascular endothelium leading to platelet activation and thrombosis on graft reperfusion [45–47].

## Recent Developments in Pancreatic Machine Preservation

The relative success in kidney and liver MP has renewed interest in pancreatic MP over the past few years, and there have been several recent reports of experimental models using HMP and NMP (see Table 1).

**Table 1:** Summary of recent studies of pancreatic machine perfusion

Author	Country	Model type	Perfusion protocol	Outcome/viability measures	Conclusion
Hypothermic machine perfusion studies					
Hamaoui [48•]	UK	Porcine DCD and human DBD pancreas	Low pressure with UW solution for 5 h then normothermic reperfusion to assess viability	During HMP: perfusion dynamics, histology, microdialysis; during normothermic reperfusion: perfusion dynamics, insulin secretion, exocrine secretion, histology, microdialysis	Successful perfusion at low pressures demonstrated no deleterious histological or oedematous effect; reperfusion assessment demonstrated viable stable perfusion and evidence of maintained insulin secretion to glucose stimulus and endocrine viability.
Leemkuil [49]	Netherlands	Human DCD and DBD pancreas	Low pressure with UW MPS for 6 h, active oxygenation	Cellular ATP, histology, ROS generation, islet isolation and culture	Oxygenated HMP regenerated cellular ATP levels, minimal histological evidence of ROS generation or apoptosis, 90% islet viability after isolation and culture
Branchereau [50]	France	Human DBD pancreas	Low pressure with UW MPS for 24 h	Macroscopic appearance, perfusion dynamics, histology	Extended duration HMP with improving resistance index during HMP, minimal macroscopic oedema, minimal cellular oedema or necrosis (vs SCS control), positive immunohistostaining for insulin and glucagon in islets in HMP preserved pancreata.
Normothermic machine perfusion studies					
Barlow [51]	UK	Human DCD and DBD pancreas	55 mmHg perfusion, ABO-compatible blood perfusate for 2 h	Macroscopic appearance, perfusion dynamics, insulin levels, acid-base balance, histology	Feasible application of strategies used in kidney NMP to the pancreas with stable perfusion dynamics, maintenance of pH homeostasis, but heterogeneous insulin secretion and focal and patchy acinar and fat necrosis, demonstrating need for further development.
Nassar [52]	USA	Human DBD pancreas	60 mmHg perfusion with ABO-compatible blood perfusate for 6-12 h	C-peptide level, histology, immunohistostaining for islets	In this limited study, after an extended duration of NMP, histological assessment demonstrated limited necrosis, and perfusate C-peptide levels increased during perfusion suggesting viability.
Kuan [53]	Australia	Porcine DCD pancreas (+ kidney)	Dual perfusion of kidney/pancreas vs pancreas alone, with autologous whole blood for 2 h at 70 mmHg	Perfusion dynamics, acid-base balance, macroscopic appearance, histology	A short duration of NMP using an autologous whole blood demonstrated stable perfusion maintenance of acid-base homeostasis, but both macroscopic and microscopic evaluations demonstrated severe oedema, haemorrhagic congestion, acinar necrosis, and vessel thrombosis by 90 min.
Kumar [54]	UK	Porcine DCD pancreas	Perfusion with autologous whole blood for 4 h comparing low (20 mmHg) or high (50 mmHg) pressure	Perfusion dynamics, acid-base balance, insulin secretion, exocrine function, macroscopic appearance, histology	Both pressures produced stable flow and equal oxygen consumption, but there was a global deterioration in acid-base haemostasis. Exocrine functionally was similar, but greater insulin release in the high-pressure group. Conversely, there was improved cell death profile, and greater ATPase activity with low-pressure perfusion.

### Pancreatic Hypothermic MP

**Fig. 2:**
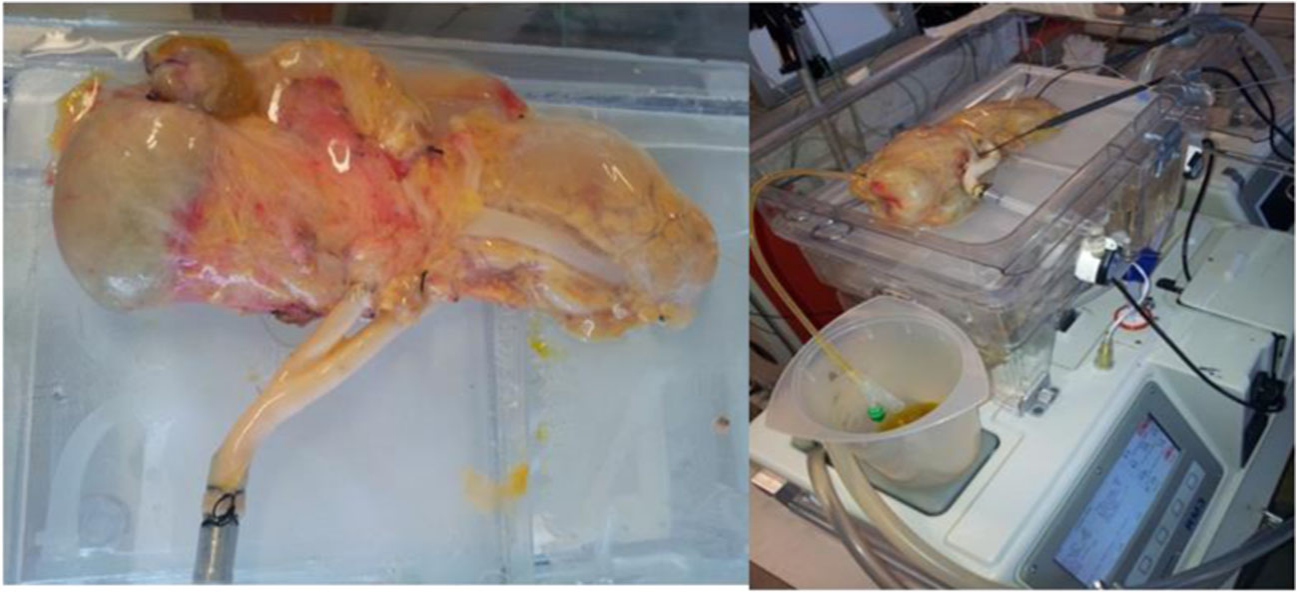
Photo of human pancreas graft undergoing hypothermic machine perfusion and subsequent normothermic reperfusion viability assessment. (Reprinted from Hamaoui et al., with permission from Elsevier) [48•]

At Imperial College London, we successfully demonstrated in 13 pancreata that HMP using low pressures with UW solution, on a modified renal perfusion system (Waters Medical System, RM3), is successful in preservation of a whole pancreas graft [48•]. This translational model was developed and refined in porcine pancreata and applied successfully to human donor pancreata allocated for research (see Fig. 2). Five hours of conventional passively oxygenated HMP at low pressures enabled stable perfusion, no deleterious oedema, and comparative histological changes to SCS controls. After preservation, viability assessment using an oxygenated normothermic reperfusion circuit demonstrated preservation of perfusion dynamics, cellular architecture, and both exocrine and endocrine functionality, with increased insulin secretion to a glucose stimulus. The study demonstrated both the technical feasibility of pancreatic HMP using a conventional clinically approved perfusion device and provided evidence of minimal deleterious effects to pancreatic architecture and function.

Leemkuil et al. from Gröningen in the Netherlands recently reported experimental HMP for 6 h in 20 human DCD and DBD donor pancreata at similar low pressures using an in-house oxygenated perfusion circuit [49]. They found oxygenated HMP regenerated cellular ATP levels compared with SCS controls, resuscitating the levels in DCD pancreata to that of DBD pancreata. There was minimal histological evidence of ROS generation or apoptosis, and though they did not assess functional pancreatic graft viability via a reperfusion circuit, two pancreata after HMP underwent islet isolation, which demonstrated 90% viability after 3 days in culture.

More recently, Branchereau et al. from the Nantes group in France evaluated the feasibility of an extended duration of 24 h of HMP in 11 human pancreata [50]. All pancreata were perfused at low pressures on a conventional kidney perfusion machine (Waters Medical System, Waves). The extended low-pressure HMP was associated with an improving resistance index during perfusion, minimal subjective visible oedema, no histological evidence of cellular oedema or necrosis (compared with SCS control pancreata where patchy necrosis was evident), and positive immunohistostaining for insulin and glucagon in islets in HMP-preserved pancreata.

### Pancreatic Normothermic MP

In parallel to the interest in HMP preservation, the exciting developments in NP in kidney and liver preservation have fueled attempts in implementing such strategies in the pancreas.

Barlow et al. from the Cambridge UK group were the first to attempt the application of ex vivo normothermic perfusion borrowed from their successful work in the kidney to five deceased donor pancreata retrieved but not used for clinical transplantation [51]. Pancreata were perfused after a 13–18 h of CIT for 120 min at 50–55 mmHg at normothermic temperatures using oxygenated ABO-compatible packed red blood cells (PRBC). One pancreas with a previous CIT of > 30 h was excluded from analysis due to macroscopic organ ischaemia during NP. In the remaining 4, there was stable perfusion dynamics with maintenance of pH homeostasis. Pancreata produced heterogeneous levels of insulin both at the start of perfusion and after stimulation by glucose and arginine, perhaps representing the heterogeneity between the donor demographics and prior ischaemic injury. Similarly, perfusate amylase and lipase levels were variable during perfusion. Histologically, all pancreata demonstrated focal and patchy acinar and fat necrosis. Though demonstrating feasible application of strategies used in kidney perfusion to the pancreas, this study highlighted the need for further development of these techniques in relation to the timing for NMP, to limit additional damage to grafts, and identify optimal viability assessment criteria.

Nasser et al. from the Cleveland Clinic reported limited success in small proof of concept study perfusing three human DBD pancreata on a modified normothermic liver perfusion circuit [52]. Pancreata experienced minimal CIT of 4 h and were perfused with PRBC at 60 mmHg for between 6 h (*n* = 2) and 12 h (*n* = 1). Histological assessment demonstrated limited necrosis, and perfusate C-peptide levels increased during perfusion. Interestingly, CIT was limited compared with the Barlow et al. study [51] and the results though limited by very small sample size may reflect this difference.

Difficulties in direct application of NMP from the kidney and liver to the human pancreas have inevitably led to further research using large animal porcine models to refine these techniques and define ideal perfusion protocols relevant to the pancreas.

Kuan et al. in Australia reported results with dual normothermic perfusion of both a porcine DCD kidney and pancreas [53]. In a novel approach, they compared the feasibility of NMP in porcine pancreas (*n* = 2) vs dual pancreas and kidney (*n* = 2), using the kidney as dialysis unit. Perfusion was with heparinised autologous whole blood on a dual perfusion roller-pump in-house based system with pancreatic perfusion pressure maintained at 70–80 mmHg and kidney at 90–100 mmHg, for 2 h. Despite minimal CIT and WIT prior to the start of NMP, stable perfusion, and maintenance of acid-base homeostasis, both macroscopic and microscopic evaluations demonstrated severe oedema, haemorrhagic congestion, acinar necrosis, and vessel thrombosis by 90 min. The authors noted severe haemolysis as perfusion progressed, and this is likely related to the pump system used. Furthermore, ex vivo porcine models utilizing autologous whole blood are more akin to ischaemia-reperfusion viability assessment models rather than preservation due to the presence of platelets, inflammatory cells, and plasma containing clotting factors and inflammatory mediators. In such settings, aggressive anticoagulation is required to ameliorate graft and circuit thrombosis. This problem is somewhat ameliorated in human pancreas models due to the use of perfusate composed of PRBC only without plasma and other normal blood constituents.

Kumar et al. from Leicester in the UK have directly compared low vs high perfusion pressures for NMP of porcine pancreata [54]. Thirteen porcine DCD pancreata retrieved from an abattoir were perfused on in-house centrifugal pump based circuit using oxygenated anti-coagulated autologous whole porcine blood for 4 h at either 20 mmHg (low) or 50 mmHg (high) pressures. WIT and CIT were short (approximately 5 min and 2 h respectively). Both perfusion pressures produced stable and flow, with higher flow in the 50 mmHg. There was equity in oxygen consumption, but a global deterioration in acid-base balance during perfusion, with accumulation of lactate; however, it is unclear if oxygenation of the blood perfusate included CO_2_ normally in a 95% O_2_/5% CO_2_ ratio to enable acid-base buffering. Exocrine function via perfusate amylase demonstrated significantly lower levels in the lower perfusion group, but with similar production volume of pancreatic effluent. Endocrine function as a measure of basal insulin and release in response to a glucose stimulus demonstrated higher levels in the 50 mmHg pressure group. Immunohistochemical analysis demonstrated an improved cell death profile (Anti-Caspase 3 and M30 CytoDEATH), and ATPase activity in the low pressure perfused pancreata. Together, the amylase levels and histological data suggest improved viability with lower perfusion pressures and paradoxically higher insulin levels seen in the higher pressure groups may simply represent more aggressive washout of the graft secondary to greater shear stress and damage sustained at the higher pressures.

## Where Is Pancreatic MP Heading?

After interrogating studies of hypothermic and normothermic MP in whole-organ pancreata, it is evident these preservation strategies are promising but still require further refinement. HMP appears at this early stage of development to better promote viability compared with simple SCS, though several important questions remain.

1. What would be the most optimal timing of HMP? The relevance of continuous HMP after retrieval until transplantation vs interrupted or end ischaemic HMP is a matter for discussion. This discussion first promulgated after the conflicting findings of the Eurotransplant MPT trial (continuous HMP) [55] and the UK machine perfusion trial (interrupted HMP after SCS) [56]. Since then, animal studies suggest end ischaemic HMP may be beneficial [57]. Gallinat et al. [58] in Essen, Germany, recently report on an observational study investigating end ischaemic HMP vs SCS in 86 paired ECD DBD kidneys. HMP after a period of SCS was a multivariate factor in reducing DGF risk and suggests such a strategy may promote resuscitation of organ viability. In the context of pancreas transplantation, due to the susceptibility of the organ to immune rejection, it would be important to determine after what degree or duration of SCS that even HMP would be unable to restore viability.
2. Status of the active provision of oxygen during HMP: the potential benefit of active oxygenation of the perfusate during HMP is currently being investigated in kidney and liver HMP. Experimental large animal studies in kidney and liver have suggested active oxygenation is required to fully realize the benefits of dynamic perfusion [59]. The Consortium for Organ Preservation in Europe is currently investigating the benefits of oxygenated HMP in two clinical trials due to end this year (ISRCTN63852508 and ISRCTN32967929).
3. How do we determine graft viability? A comprehensive approach utilizing HMP would include viability assessment at the start of preservation (after SCS or immediately after retrieval) and determination if there is an improvement during preservation (suggestive of resuscitation), with correlation of these viability profiles to outcomes including manifestations of ischaemia-reperfusion (IR) injury such as delayed function, graft pancreatitis, and thrombosis. Current measures used in the few experimental trials include consideration of hypothermic perfusion profiles and histological assessments of tissue damage [48•, 49, 60]. Specific perfusate biomarkers have not been identified—though markers under investigation in kidney MP include markers such as glutathione S-transferase, lactate dehydrogenase, heart-type fatty acid binding protein, redox-active iron, interleukin 18 (IL-18), and neutrophil gelatinase-associated lipocalin (NGAL) [33]. Current evidence suggests that a composite of viability metrics together rather than an isolated marker alone would be the most beneficial in determining organ viability. With the recent breakthroughs in metabolomics, proteomics, and genomics, focus on grafts from a bio-cellular and metabolic perspective during preservation may be the most revealing determinant of viability—examining metabolic function rather than damage. In our unit, we believe direct assessment of tissue metabolism is key to determining the recuperative potential of any organ undergoing dynamic preservation. Real-time microdialysis is a non-invasive technique that allows for the interstitial composition of a target tissue to be sampled, followed by ex vivo analysis, providing an accurate method of monitoring tissue biochemistry and metabolism over time. This is one technology that is being trialled in clinical and experimental settings of tissue preservation [61].
4. The potential for therapeutic interventions during HMP ameliorating immune rejection injury and potentially postoperative graft dysfunction: our group recently showed that thrombalexin—a cell membrane tethering derivative of the direct thrombin inhibitor hirudin—adheres to the endothelium in both porcine and human kidneys when administered during HMP [62]. In a haemoreperfusion model, presence of these peptides improved both micro- and macrovascular organ perfusion and ameliorated intra-graft microvascular coagulation. The potential benefit in ameliorating graft microvascular endothelial dysfunction in pancreatic grafts is high, due to the emergent understanding of the complex interface between IR injury and microvascular coagulopathy, and progression to macrovascular graft thrombosis and pancreatitis—the leading cause of early graft loss.

As an alternative to HMP, the development of NMP has the greatest potential to offer a physiologically optimal environment to counter the deleterious effects of SCS CIT before transplantation. Ischaemia-reperfusion and graft injury can be controlled in an environment without the inflammatory milieu encountered at transplantation. This would allow stabilization of the graft biochemically, histologically, and potentially functionally and would enable both assessment of organ viability and also allow intervention. Extrapolation from kidney and liver MP suggests this is achievable.

Similar questions to those posed for the development of HMP exist however relating to timing of NMP, the extent to which NMP can resuscitate grafts, the optimal way to determine graft viability, and the potential for intervention. Assessment of graft function should involve the interrogation of cellular metabolism (oxygen consumption, ATP generation, acid-base homeostasis), cellular architecture (apoptosis), macroscopic oedema, microvasculature disruption (endothelial inflammation and thrombosis), and both endocrine and exocrine functionality.

Recent reports have described interventions during NMP in kidney, which may prove useful in optimising high-risk grafts once stable models of pancreatic NMP have been established and validated—rendering grafts previously considered untransplantable into viable options for patients.

The use of novel localizing agents to target graft vascular endothelial has been described as immuno-camouflage [62]. This strategy not only presents opportunities to target anti-coagulant [62, 63] and anti-inflammatory therapeutics to the graft itself which will confer a localized and potentially optimal effect compared with systemic administration after transplantation, but also to potentially repair the ischaemically damaged endothelium [64] and limit immunogenicity [65]. MP is thus an ideal delivery mechanism, and such strategies would be beneficial in the pancreas which is highly susceptible to IR microvascular injury, the inflammatory milieu that ensues, and then subsequent thrombosis.

In addition to the provision of oxygen, MP can facilitate exposure of organs to other specific gases. Certain gases in experimental animal models have been shown to beneficially ameliorate IR injury—such as CO [66], hydrogen sulphide [67], and nitrous oxide [68]. MP through either introduction of novel gases into the circuit and dissolution in the perfusate or through addition of specific molecular donors would potentially allow conditioning of grafts, and amelioration of IR injury is a further avenue of research being explored.

Progress in gene therapies introduces the prospect of altering gene expression in donor organs, and modifying organs ex vivo introduces the potential for more effective and personalized medicine therapies in transplantation [69]. Adenovirus vector delivery has been successful in ex vivo lung models [70], and with the progress in therapeutics in RNA interference aiming to modulate IR related genes, there have been isolated promising reports in the kidney and liver [71, 72]. In particular, the advent of the first RNA interference drug gaining US Food and Drug Administration approval for clinical use opens the path for further development and MP is an ideal delivery mode for such drugs for the ex vivo modification of donor organs [73].

Strategies to re-condition and rejuvenate marginal organs during MP have explored the use of mesenchymal stem cells (MSCs) for their anti-inflammatory properties and regenerative capacity [74]. The promise of MSC during MP is the amelioration of IR injury and its effects by attenuating inflammatory processes, regenerating injured tissue, and with their presence in the graft after transplantation dampening the immune response, aiding with acute rejection and graft function.

## Conclusion

The next most important development in organ transplantation will amalgamate and incorporate isolated preservation strategies using MP into regional organ assessment and repair centres (ARC) [75]. Organs from donors considered high risk based on donor risk indices could be retrieved, stored via SCS, and transported to a regional ARC, where they would undergo MP, assessment of organ quality, viability, and function. After assessment, a period of optimisation, and if required intervention and repair (pharmacological therapeutics) or modification (gene expression modification, or MSC administration), could be instituted, before a final decision is made on suitability of transplantation.

Further development and refinement of both HMP and NMP and specific interventions are still required; however, based on the trajectory of the rapid progress made in dynamic organ preservation over the past decade, these are fast becoming a reality.

In such circumstances, optimisation of high-risk organs and restoring viability and functional capacity to a level considered acceptable by clinicians for transplantation would expand the donor pool by a subset of donor organs previously discarded. If this can increase the conversion rate of retrieved to transplanted pancreatic grafts in the UK by 10%, this could portend a further 50 donor transplants a year; if the conversion of offered to retrieved organs also increased by 10%, this would make a further 80 donor organs available in the donor pool. In the USA where the majority of pancreas transplants are from DBD donors and there is a 20% discard rate [76–78], a decline in this figure by a modest 25% would result in 70 more transplants a year a 7% increase overall. Furthermore, if there is also renewed acceptance of DCD donor pancreata increasing from 3% at present closer to the rates in the UK or Europe (approximately 20% [77]), such an expansion could equate to 200 more transplants per year. In both situations, the availability of more suitable donors determined by objective measures during dynamic preservation and optimisation does need to be matched by clinicians and patients who are willing to accept the recent successful results and proven benefits of a pancreas transplant on both survival and quality of life. Such a shift must come from a collaborative of improved peer-education, national diabetes and patient organizations, and from transplanted patients themselves who can testify to these achievements.
